# Using an Isolated Rat Kidney Model to Identify Kidney Origin Proteins in Urine

**DOI:** 10.1371/journal.pone.0066911

**Published:** 2013-06-25

**Authors:** Lulu Jia, Xundou Li, Chen Shao, Lilong Wei, Menglin Li, Zhengguang Guo, Zhihong Liu, Youhe Gao

**Affiliations:** 1 Department of Physiology and Pathophysiology, National Key Laboratory of Medical Molecular Biology. Institute of Basic Medical Sciences, Chinese Academy of Medical Sciences/Peking Union Medical College, Beijing, China; 2 Research Institute of Nephrology, Jinling Hospital, Nanjing University School of Medicine, Nanjing, China; Universidade de Sao Paulo, Brazil

## Abstract

The use of targeted proteomics to identify urinary biomarkers of kidney disease in urine can avoid the interference of serum proteins. It may provide better sample throughput, higher sensitivity, and specificity. Knowing which urinary proteins to target is essential. By analyzing the urine from perfused isolated rat kidneys, 990 kidney origin proteins with human analogs were identified in urine. Of these proteins, 128 were not found in normal human urine and may become biomarkers with zero background. A total of 297 proteins were not found in normal human plasma. These proteins will not be influenced by other normal organs and will be kidney specific. The levels of 33 proteins increased during perfusion with an oxygen-deficient solution compared to those perfused with oxygen. The 75 proteins in the perfusion-driven urine have a significantly increased abundance ranking compared to their ranking in normal human urine. When compared with existing candidate biomarkers, over ninety percent of the kidney origin proteins in urine identified in this study have not been examined as candidate biomarkers of kidney diseases.

## Introduction

The identification of urinary biomarkers of kidney disease may be easier to accomplish than the identification of biomarkers for other diseases such as cancer. The biomarker identification pipeline has been divided into two separate stages: discovery and validation [1]. However, despite substantial interest and investment, only a few novel urinary biomarkers are currently used in clinical practice [2]. Clinical use is limited because comprehensive, profiling-based differential proteomics methods, which have limited sample throughput because of their prolonged sample analysis, are generally used in the discovery phase [3]. Profiling is also easily influenced by the preferential detection of highly abundant proteins. As a result of this bias, the detection in urine of less abundant proteins, which are believed to be more specific, is suppressed. Furthermore, highly abundant plasma proteins, which exhibit similar changes under many different renal conditions and lack specificity, are repeatedly identified [4]. These circumstances are often aggravated by proteinuria as a comorbidity [5].

Advances in targeted proteomic technologies simultaneously allow the quantification of hundreds of proteins with better sample throughput, high sensitivity, and high specificity [6]–[8]. The disadvantages of profiling methods can be avoided by using targeted proteomic technologies in the discovery phase. The key is to target the right proteins.

Kidney origin proteins in urine include proteins that are secreted or shed by the cells and tissues of the kidney and proteins that leak into the fluid from aged or damaged tissue. Injury to different renal cells is expected to generate different proteins in urine, which may be more representative of the state of the kidney [9] and may be more readily detectable than the tumor-associated proteins that are released early in oncogenesis. Identifying quantitative changes in kidney origin protein levels in urine may yield information that is pertinent to the functions of renal cells and has a greater chance of detecting changes in renal function at an early stage. However, the identification of urinary proteins that are directly derived from the kidney is required.

To the best of our knowledge, there have been no studies that directly and comprehensively identified proteins of kidney origin in urine. An analysis of kidney origin proteins in urine should exclude proteins present in urine as the result of the ultrafiltration of plasma. Isolated kidney perfusion is a classic technique that has been widely used for the study of renal physiology, pharmacology, and pharmacokinetics [10]. A perfused isolated kidney can maintain approximately normal physiological functions for more than two hours when it is circularly perfused with artificial blood-free perfusion fluids, such as buffered saline solutions supplemented with macromolecular plasma substitutes [10].

In this study, we used a modified isolated rat kidney perfusion model to analyze the kidney origin proteins present in the urinary tract.

## Experimental Procedures

### Ethics Statement

This study was approved by the Institute of Basic Medical Sciences Animal Ethics Committee, Peking Union Medical College (Animal Welfare Assurance Number: A5518). Male Sprague-Dawley rats (350–450 g) from the Institute of Laboratory Animal Science were maintained at approximately 21°C on a 12 h light/dark cycle with free access to food and water.

### Perfusate Preparation

Modified Krebs-Henseleit buffer (4.7 mM KCl, 1.2 mM KH_2_PO_4_, 2.1 mM MgSO_4_, 117 mM NaCl, 25 mM NaHCO_3_, 2.5 mM CaCl_2_, 11 g/L glucose) was used as a perfusate with 60 g/L clinical grade dextran added as an oncotic agent [11]. Twenty amino acids were combined in solution to achieve the following concentrations: 2 mM alanine, 0.5 mM arginine, 0.2 mM asparagine, 0.2 mM aspartate, 0.5 mM cysteine, 0.5 mM glutamate, 2 mM glutamine, 2.3 mM glycine, 0.24 mM histidine, 0.3 mM isoleucine, 0.4 mM leucine, 1 mM lysine, 0.33 mM methionine, 0.32 mM phenylalanine, 0.31 mM proline, 1 mM serine, 0.24 mM threonine, 0.07 mM tryptophan, 0.2 mM tyrosine, and 0.33 mM valine [12]. The perfusate was filtered through a 0.45 µm filter and equilibrated with a mixture of oxygen and carbon dioxide (95% O_2_/5% CO_2_) for at least two hours prior to use. The perfusate was used within six hours of preparation, and the pH of the solution was adjusted to 7.4 with hydrochloric acid prior to use.

### The Isolated Rat Kidney Perfusion Model

The surgical technique was based on methods described previously [10]. Briefly, rats were anesthetized via an intraperitoneal injection of sodium pentobarbitone (40 mg/kg). A midline laparotomy incision was made from the pelvis to the sternum on the animal. The right ureter was ligated immediately proximal to the bladder. A solution of mannitol (150 mg) and heparin (100 U) in 1 ml normal saline was injected into the venae saphena magna. The right ureter was cannulated with a 24G vein detained needle to collect the urine. The aorta was clamped distal to the right renal artery and cannulated with an 18G vein detained needle a few millimeters distal to the renal artery in the retrograde direction. The superior mesenteric artery and the aorta proximal to the right renal artery were ligated so that the artificial perfusates could flow into the right kidney. The infusion tube was inserted into the inferior vena cava from the near heart end to arrive at the right renal vein to drain the perfusates. After the completion of the surgery, the rat was transferred into a small incubator to maintain a temperature of 37°C.

The isolated kidney was perfused in situ. Perfusates, aerated with a mixture of 95% O_2_/5% CO_2_, were sequentially pumped through the in-line filter, the bubble trap, and a 37°C warming system to enter the isolated kidney. The intrarenal perfusion pressure was maintained at 110±5 mm Hg by adjusting the flow rate of the rotary pump, which was continuously monitored using a manometer and was corrected for the intrinsic pressure of the apparatus.

The isolated kidney was first perfused in single pass-perfusion mode for 10–15 min, allowing the kidney to pre-equilibrate and flushing out the residual blood in the kidney. The mode was then changed to recirculating mode with a recirculating perfusion medium volume of 300 ml and a duration of 40 min. The kidney was perfused with oxygen-supplemented perfusion medium during this period. The urine was collected at 10-minute intervals during this stage. When the first stage was complete, 2 ml of the perfusates and perfusion-driven urine was centrifuged at 12,000×*g* for 10 min at 4°C to determine if there were red blood cells in the perfusates, and the cell debris in the perfusion-driven urine was evaluated by macroscopic observation. Only isolated perfused kidneys that were negative for both of the above inspections were considered to be successful preparations and proceeded to the next stage. In the next stage, another 300 ml of fresh perfusion medium was used to perfuse the kidney for another 40 min under the same conditions, except that the perfusion medium was not supplemented with oxygen. The perfusion pressure and flow rate were recorded at 10-minute intervals over both stages of the experiments. When the experiment was complete, the perfusion-driven urine with and without oxygen supplementation was collected and prepared for analysis.

### Preparation of Proteins in Perfusion-driven Urine

#### SDS-PAGE separation for the comprehensive analysis of the perfusion-driven urine with oxygen supplementation

To identify proteins in urine that were collected during perfusion with oxygen-supplemented medium, the collected urine was loaded onto 20×12-cm, 12% polyacrylamide gels for separation. After the gels were stained with colloidal Coomassie blue, the lanes were excised into 26 1–2-mm-wide slices and subjected to in-gel digestion.

#### Gel concentration for the comparison of perfusion-driven urine with and without oxygen supplementation

The combined perfusion-driven urine from one rat with or without oxygen supplementation was transferred into a 3-kDa cutoff centrifugal column to reduce the volume to 250 µL. Proteins were loaded onto a custom 12% acrylamide gel with large wells for SDS-PAGE analysis. Electrophoresis was stopped when the proteins were concentrated in bands between the stacking and resolving gels according to the prestained protein marker. The protein bands were cut into small pieces and subjected to in-gel digestion. The peptides that were extracted from these gel pieces were prepared for LC-MS/MS analysis.

### Protein Digestion and Peptide Preparation

For in-gel digestion, the protein band on the SDS-PAGE gel was cut into 1 mm^3^ pieces and treated with a destaining solution containing 50% acetonitrile in 50 mM NH_4_HCO_3_. The proteins in the gel pieces were then reduced using 10 mM DTT for 1 hour at 56°C and were alkylated with 55 mM iodoacetamide for 45 min at room temperature. The reduced and alkylated proteins were digested with sequencing-grade modified trypsin at 1∶25 wt:wt for 16 hours at 37°C in 50 mM NH_4_HCO_3_, pH 8.0. The resulting peptides were extracted twice with 5% or 2.5% TFA in 50% acetonitrile/water for 1 hour at 37°C. The two extractions were combined and filtered with a 10-kDa-cutoff centrifugal column. The flow-through solution containing peptides was dried via vacuum evaporation and resuspended in an aqueous solution containing 0.1% formic acid prior to LC-MS/MS analysis.

### LC-MS/MS Analysis

#### LTQ Orbitrap Velos platform

The tryptic peptides were sequentially loaded onto a Michrom Peptide Captrap column (MW 0.5–50 kD, 0.5 × 2 mm; Michrom Bioresources) at a flow rate of 20 µL/min in 0.1% formic acid/99.9% water. The trap column effluent was then transferred to a reversed-phase microcapillary column (0.1 × 150 mm, packed with Magic C18, 3 µm, 200 Å; Michrom Bioresources) in an Agilent 1200 HPLC system. Peptide separation was performed at 500 nL/min and was coupled to online analysis using tandem MS with an LTQ Orbitrap Velos (Thermo Fisher Scientific, San Jose, USA). The elution gradient for the reverse column changed from 95% mobile phase A (0.1% formic acid, 99.9% water) to 40% mobile phase B (0.1% formic acid, 99.9% acetonitrile) within 120 min. The MS was programmed to acquire data in data-dependent mode. MS survey scans were acquired using an Orbitrap mass analyzer; the lock mass option was enabled for the 445.120025 ion, and MS/MS were analyzed in the LTQ. The MS survey scan was obtained over an *m/z* range of 300–2000 (1 μ scan) with a resolution of 60000 and was followed by twenty data-dependent MS/MS scans (1 μ scan, isolation width of 3 *m/z*, dynamic exclusion for 0.5 min). The 20 most intense ions were fragmented in the ion trap by collision-induced dissociation with a normalized collision energy of 35%, an activation q value of 0.25 and an activation time of 10 ms.

#### TripleTOF 5600 Platform

The tryptic peptides were analyzed using an RP C18 capillary LC column from Michrom Bioresources (100 µm×150 mm, 3 µm). The eluted gradient was 5–30% buffer B (0.1% formic acid, 99.9% ACN; flow rate, 0.5 µL/min) for 100 min. MS data were acquired in the TripleTOF MS using an ion spray voltage of 3 kV, curtain gas of 20 PSI, nebulizer gas of 30 PSI, and an interface heater temperature of 150°C. The precursor scans ranged from 350 to 1250 m/z and were acquired over 500 ms; the product ion scans ranged from 250 to 1800 m/z and were acquired over 50 ms. A rolling collision energy setting was used. In total, 30 product ion scans were collected that exceeded a threshold of 125 counts/s with a +2 to +5 charge-state for each cycle.

### Database Searching and Protein Identification

All of the MS/MS spectra were searched against the rat IPI 3.87 protein database using MASCOT 2.4.0. The search parameters were set as follows: tryptic cleavages at only lysine or arginine with up to two missed cleavage sites allowed; fixed cystein carbamidomethylation; variable aspartic acid and glutamine deamidation; and variable methionine oxidation. For MS files acquired from the LTQ Orbitrap Velos, the precursor mass tolerance was set to 10 ppm and the fragment mass tolerance to 0.5 Da. For MS files acquired from the TripleTOF 5600, the precursor mass tolerance was set to 0.05 Da and the fragment mass tolerance to 0.05 Da.

### Enrichment Analysis of Gene Ontology Categories

BiNGO, a Cytoscape plug-in, was used to find statistically overrepresented GO categories [13]. The whole human release of the UniProt-GOA Database, available from the EBI website, was used as a reference dataset. The human kidney origin proteins in urine were performed the enrichment analysis. The analysis was performed using the “hyper geometric test”, and all GO terms that were significant (*P*<0.001) after correcting for multiple term testing using the Benjamini and Hochberg false discovery rate correction were selected as overrepresented.

## Results

### 1. SDS PAGE Analysis of the Perfusion-driven Urine

**Figure 1:**
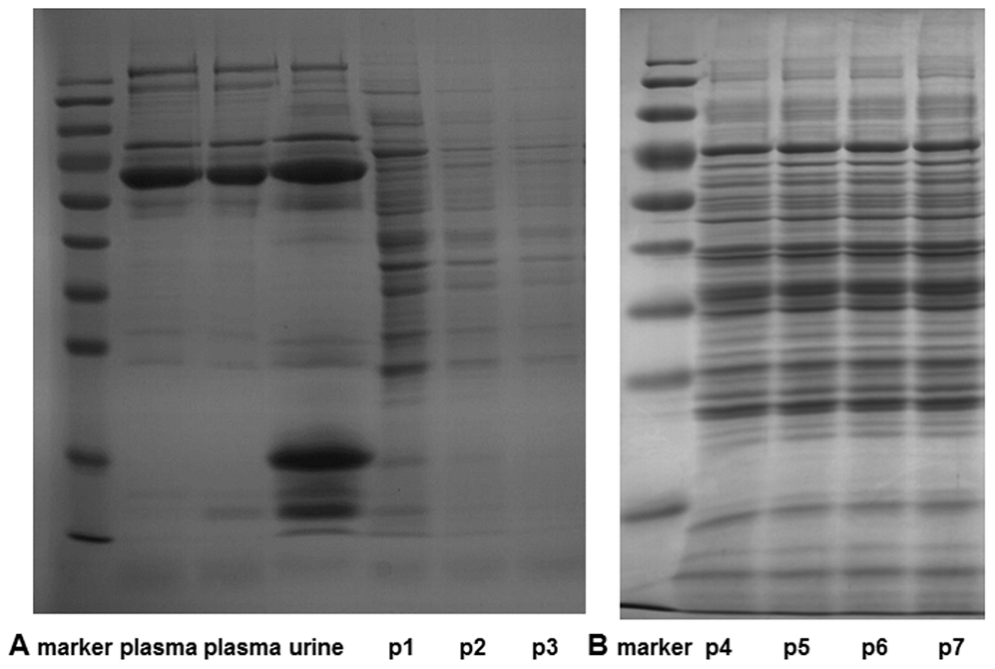
SDS-PAGE analysis of perfusion-driven urine. (A) The proteins from the perfusion-driven urine with oxygen supplementation were resolved and compared with the proteins present in rat plasma and rat urine. Lane p1, p2, and p3 represents proteins acquired from the first, second, and third ten-minute intervals of the perfusion respectively (B) The proteins from the perfusion-driven urine without oxygen supplementation were resolved. Lane p4, p5, p6, and p7 represents proteins acquired from the first, second, third, and fourth ten-minute intervals of the perfusion respectively.

The proteins in the perfusion-driven urine were separated using SDS-PAGE. Equal volumes of the perfusion-driven urine were loaded. As shown in Figure 1A, the proteins present in the perfusion-driven urine were quite different from those in either the plasma or urine. There was no apparent difference in the proteins present in the perfusion-driven urine with and without oxygen supplementation, which may be due to the poor resolving power of SDS-PAGE (Figure 1B). In the perfusion-driven urine with oxygen supplementation, the concentration of the proteins decreased as perfusion continued; this decrease was not observed in the perfusion-driven urine without oxygen supplementation. The protein concentration of the perfusion-driven urine without oxygen supplementation was much higher than that of the perfusion-driven urine with oxygen supplementation, which suggests that there may have been kidney injury due to the lack of oxygen.

### 2. Comprehensive Profiling of the Perfusion-driven Urine Proteome using SDS-PAGE-LC-MS/MS

#### 2.1 Identification of proteins in the isolated rat kidney perfusion-driven urine

The proteins present in perfusion-driven urine were separated using SDS-PAGE. Lanes were cut into twenty-six slices. After digestion of the proteins with trypsin, each slice was analyzed using LC-MS/MS. MS/MS files acquired from each fraction were merged, and the proteins were identified by performing a database search using MASCOT.

Two perfusion-driven urine samples acquired from two independent isolated rat kidneys were analyzed using different mass spectrometry platforms, an LTQ Orbitrap Velos platform and a high speed TripleTOF 5600 system. A total of 1,782 and 3,025 proteins, respectively, were identified with more than two distinct peptides (Table S1). There are 1,402 proteins common to both samples. The proteins common to both methods were subjected to subsequent analysis.

#### 2.2 Identification of human orthologs for the proteins in isolated rat kidney perfusion-driven urine

This study aims to find human kidney origin proteins in urine. It is typically assumed that orthologs (co-orthologs) retain similar functions between species [14], [15]. Therefore, we identified human orthologs for proteins in the isolated rat kidney perfusion-driven urine. However, there is currently no “gold standard” for identifying a complete set of orthologs between two species [16]. Different orthologous protein databases use the different orthology prediction methods and thus yielded different and overlapping results. InParanoid [17], OrthoMCL-DB [18], Homogene [19], and Ensembl Compare [20] are four well-known databases that contain such information. An additional database, 122.R_norvegicus.orthologues, is found on the EBI website (http://www.ebi.ac.uk/). We used these five databases to search for human orthologs of identified proteins. From the 1,402 rat proteins, 1,055, 1,096, 1,150, 1,180, and 937 proteins were matched to human orthologs according to the InParanoid [17], OrthoMCL-DB [18], Homogene [19], Ensembl Compare [20], and 122.R_norvegicus.orthologues databases, respectively. Human orthologs that were identified from the same rat proteins by at least two databases were compiled, resulting in the pairing of 1,234 of the 1,402 rat proteins to 1,233 human orthologs, which account for 1,278 human orthologous genes.

We analyzed the data for human orthologs, and we propose that in biomarker studies that use animal models, it is best to choose proteins that have human orthologs to facilitate the translation of the data into human applications.

#### 2.3 Comparison of human orthologs for proteins in the perfusion-driven urine with kidney protein expression data, the normal human urine proteome, and the plasma proteome

While the human orthologs expressed in the kidney are likely to be kidney origin proteins found in the normal human urine, the human orthologs not expressed in the kidney may be residual interstitial fluid proteins and/or may be plasma proteins that were absorbed by the kidney. To identify kidney origin proteins in urine, the human orthologs were compared with human kidney expression data. Data detailing the expression of human kidney proteins were acquired from the Human Protein Atlas Database [21], which was constructed to show the expression and localization of proteins in a variety of normal human tissues. Expression data from 12,260 human kidney genes were acquired from the Human Protein Atlas Database [21].

The human orthologs of rat perfusion-driven urine proteins were also compared with the normal human urine proteome (including the urinary exosome proteome) and the human plasma proteome to determine which human orthologs have been identified in normal human urine and plasma, respectively. For the normal human urine proteome, three large-scale datasets from previous studies [22]–[24] and one large-scale dataset from another team at our institution (data not published) were collected. For the normal human urinary exosome proteome, three large-scale human urinary exosome datasets from previous studies were collected [25]–[27]. For the normal human plasma proteome, the largest human plasma proteome dataset was acquired from an online database, Healthy Human Individual’s Integrated Plasma Proteome (HIP^2^) [28].

For easier comparison, the protein identifiers in different datasets and the kidney expression genes were standardized. We used Ensembl BioMart (http://asia.ensembl.org/biomart/martview) to transform all of the different protein identifiers to Ensembl Gene ID(s). We compared the different proteome datasets at the gene level. All of the human urine proteins, urinary exosome proteins, and plasma proteins from different datasets were pooled together. This process resulted in 5,225 non-redundant genes in human urine, 3,416 non-redundant genes in the human urine exosome, and 9,706 non-redundant genes in human plasma. The genes in human urine and the urine exosome were pooled, which resulted in 6,084 non-redundant genes in normal human urine and the urinary exosome.

**Figure 2:**
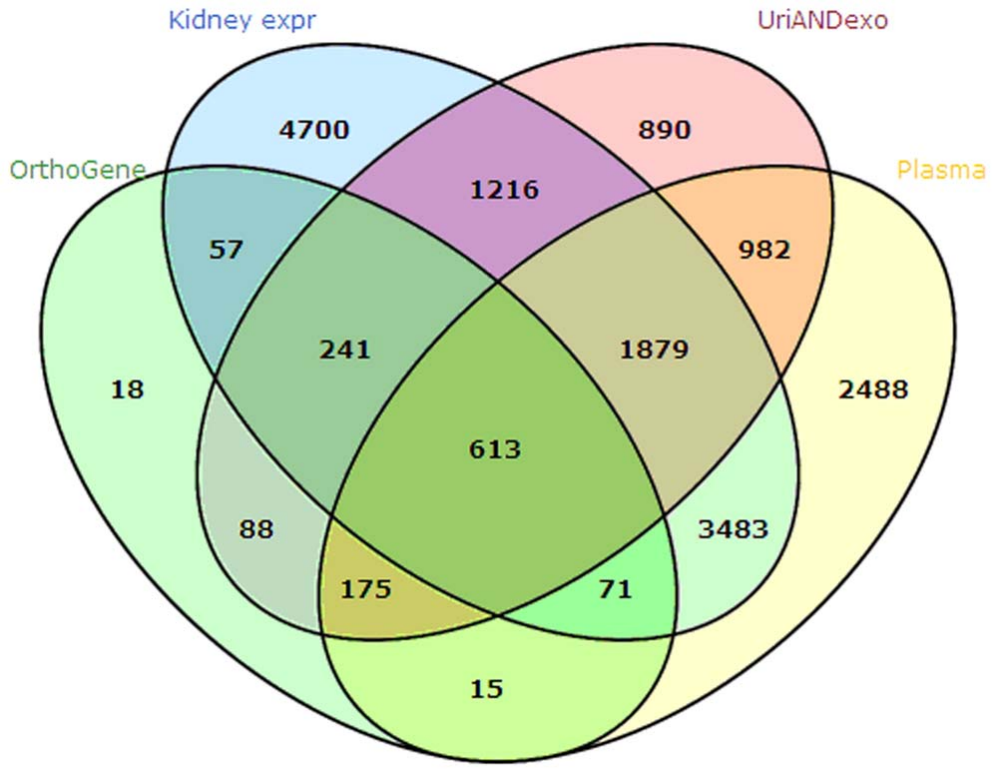
The human orthologs identified from the rat proteins in perfusion-driven urine were compared with human kidney expression data (Kidney expr), the pooled human urine and urinary exosome proteome (UriANDexo), and the human plasma proteome (Plasma). The protein identifiers were standardized using the Ensembl Gene ID(s). The comparison was performed at the gene level.

The 1,233 human orthologs, which account for 1,278 human orthologous genes, were compared at the gene level with human kidney gene expression, the pooled human urine and urinary exosome proteome, and the human plasma proteome (Figure 2).

Of the 1,278 genes, 982 were expressed in the kidney. These genes corresponded to 981 human orthologs. The 981 human orthologs with gene expression in the kidney were considered to be potential human kidney proteins in urine (Table S2). Of the 981 human orthologs, 613 had been identified both in the urine (including urinary exosome) proteome and the plasma proteome; 240 had only been identified in the urine (including urinary exosome) proteome but not in the plasma proteome; 71 had only been identified in the plasma proteome but not in the urine (including urinary exosome) proteome; and 57 had not been identified in either the urine (including urinary exosome) proteome or the plasma proteome (Figure 2).

There are a total of 128 human orthologs (57 plus 71) that were expressed in the kidney but were not present in normal urine (including the urinary exosome). They are potential biomarkers with zero background in pathological conditions. There are a total of 297 human orthologs (57 plus 240) that were expressed in the kidney but were not present in the plasma. They are likely not influenced by other normal organs, including the plasma, and therefore have the potential to specifically reflect functional changes in the kidney. The 57 human orthologs could be sensitive markers because they were not present in normal urine or the urinary exosome and were not influenced by other normal organs, including plasma.

#### 2.4 Comparing the ranking of human kidney origin proteins in the normal and perfusion-driven urine

A large-scale dataset of the human normal urine proteome has been provided by another team at our institution (data not published). They used the same TripleTOF 5600 system and the same MASCOT search engine as in this study. The Exponentially Modified Protein Abundance Index (emPAI), which offers approximate, label-free, relative quantitation of the proteins in a mixture based on protein coverage by peptide matches, has been incorporated into the MASCOT search engine [29]. Therefore, each identified urine protein had an emPAI value, which can be used to approximately estimate the absolute protein contents in urine.

Of the 981 human orthologs that were considered to be potential human kidney origin proteins in urine, 775 were identified in this normal human urine dataset. The emPAI values of these human orthologs were extracted from the normal human urine proteome, and these proteins were sorted from most to least abundant in the normal human urine. Proteins not identified in the human urine were at the end. The order of these human orthologs approximately represents their abundance in human urine under physiological conditions.

The 981 human orthologs were paired to rat proteins that were identified in both isolated rat kidney perfusion-driven urine samples. The rat proteins corresponding to human orthologs had an emPAI value when they were identified in the perfusion-driven urine, which can be used to approximately estimate the absolute protein content in the perfusion-driven urine. The rat proteins corresponding to the human orthologs were sorted according to their emPAI values in the two perfusion-driven samples from most to least abundant in the perfusion-driven urine. Due to the correspondence between rat proteins and their human orthologs, this resulted in the re-ordering of the 981 human orthologs. We assume that the abundances of orthologous proteins in the human and rat samples have a certain correlation. The new order of the 981 human orthologs sorted by the abundance of their paired rat proteins in the perfusion-driven urine might approximately represent the anundance order in the pathological condition.

For a given protein, if the abundance ranking increased significantly from the normal urine to the perfusion-driven urine, expression of that protein might increase under pathological conditions compared to other proteins. The ranks of the corresponding rat proteins in the two perfusion-driven urine samples were compared first. The vast majority, 922 proteins (94%), had ranking changes of less than 300. Therefore, a ranking change of 300 was considered to be significant. In total, 75 of the 981 human orthologs increased in rank by 300 from the normal human urine to the two perfusion-driven urine samples (Table S2).

The emPAI value is only an approximate estimation of the absolute protein content in a protein mixture [29]. The degree of correlation between orthologous protein abundance was not investigated. Here, we only observed that, for the 75 human orthologs, the rank of their corresponding rat proteins increased significantly in the perfusion-driven urine compared to their rank in the normal urine. We expect the large difference in the abundance ranking of these proteins will indicate their potential to be kidney disease biomarkers.

These 75 proteins were compared with the pooled human urine and urinary exosome proteome and the human plasma proteome. Of these proteins, 35 had been identified both in the urine (including urinary exosome) proteome and the plasma proteome; 15 proteins had only been identified in the urine (including urinary exosome) proteome but not in the plasma proteome; 13 proteins had only been identified in the plasma proteome but not in the urine (including urinary exosome) proteome; and 12 proteins had not been identified in either the urine (including urinary exosome) proteome or the plasma proteome.

### 3 Comparison of the Perfusion-driven Urine Proteomes during Perfusion with and without Oxygen Supplementation using LC-MS/MS

The urine proteomes from four independent isolated perfused rat kidneys during perfusion with and without oxygen supplementation were profiled using LC-MS/MS. The samples from two of the rats were profiled with the LTQ Orbitrap Velos platform, which identified 236 and 280 proteins during perfusion with oxygen supplementation and 275 and 281 proteins during perfusion without oxygen supplementation. The samples from the other two rats were profiled with the TripleTOF 5600 platform, which identified 474 and 466 proteins during perfusion with oxygen supplementation and 511 and 527 proteins during perfusion without oxygen supplementation.

The expression of the proteins present during perfusion with oxygen-supplemented medium was compared with expression during perfusion without oxygen supplementation using the label-free quantitative method provided by the SCAFFOLD program. The expression of 39 proteins was significantly increased in all four perfusion-driven urine samples when the kidneys were perfused without oxygen supplementation (*p*<0.05, T-test) (Table S2).

These 39 proteins were matched to human orthologs using the same method described above. In total, 33 human orthologs were identified. Because their corresponding rat proteins were increased in the perfusion-driven urine when the kidneys were perfused without oxygen supplementation, these 33 human orthologs were also considered to be the potential human kidney origin proteins in urine.

### 4 Comparison of the Human Kidney Origin Proteins in Urine with Previous Biomarker Studies

A total of 990 non-redundant human orthologs were generated by pooling the perfusion-driven urine proteins that are expressed in the kidney and increased in perfusion-driven urine from oxygen-deficient kidneys. These proteins are potential human kidney origin proteins in urine (Table S2). Of the 990 kidney origin proteins, there are a total of 428 proteins that may be high-quality potential candidate biomarkers, including kidney origin proteins present in the perfusion-driven urine but not in normal urine, kidney origin proteins present in the perfusion-driven urine but not in the large-scale plasma database, kidney origin proteins that are increased in perfusion-driven urine from oxygen-deficient kidneys, and kidney origin proteins that have a large increase in rank in the perfusion-driven urine compared to normal human urine.

**Figure 3:**
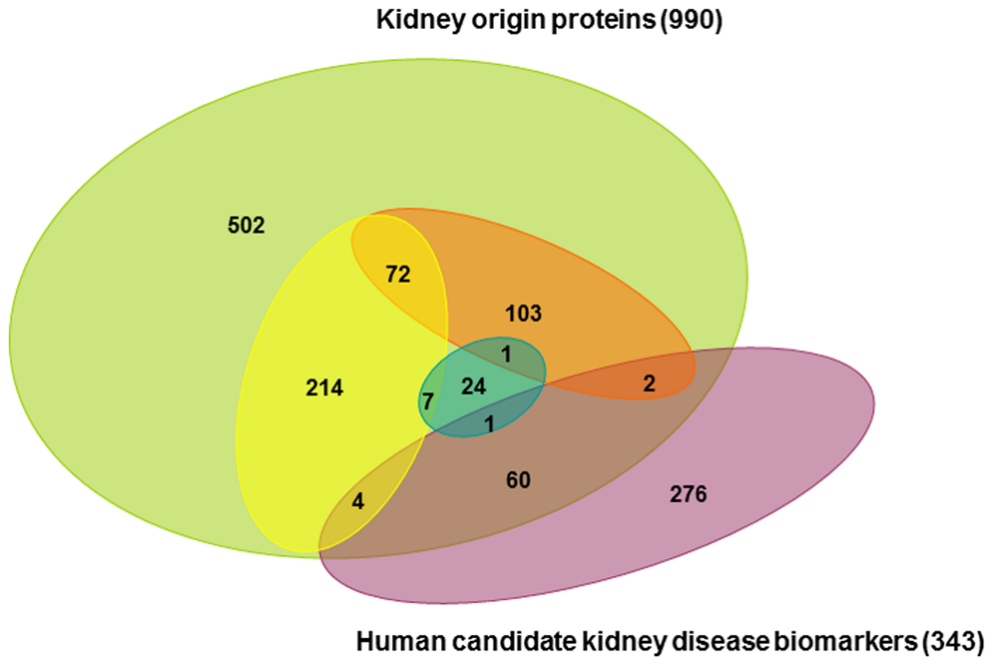
A comparison of the identified kidney origin proteins with previously identified human candidate biomarkers of kidney disease. The yellow oval represents proteins present in perfusion-driven urine but not in normal human plasma. The orange oval represents proteins detected in perfusion-driven urine but not in normal human urine (including human urinary exosomes) or present in human urine but significantly increased in the perfusion-driven urine. The blue oval represents proteins with an increased level in perfusion-driven urine without oxygen supplementation compared to perfusion with oxygen-supplemented medium.

The urinary Protein Biomarker Database was established by comprehensively compiling and manually curating the published literature [30]. A total of 343 candidate biomarkers for human kidney diseases have been collected from the Urinary Biomarker Database [30]. Compared with this database, 67 of the 990 kidney origin proteins have been studied as candidate biomarkers of kidney diseases (Figure 3). Of the 428 high-quality kidney origin proteins, 7 proteins have been studied as the candidate biomarkers of kidney diseases.

However, 923 (93%) kidney origin proteins have not been studied as candidate biomarkers. Furthermore, few of the 67 kidney origin proteins that were identified as candidates in large-scale differential proteomics experiments have been examined in more detail according to the urinary biomarkers database. One reason why studies examining urinary biomarkers in kidney disease have not been conclusive might be because kidney origin proteins were not examined in detail. These 428 high-quality kidney origin proteins are potential urinary kidney disease markers that should be examined in detail. Because there are hundreds of potentially useful urinary kidney disease markers, combinations of these proteins are likely to be able to differentiate many different kidney conditions.

### 5 Gene Ontology Annotation of Human Kidney Origin Proteins

The human kidney origin proteins in urine were classified in terms of molecular function and biological process using the BiNGO software package [13]. Candidate biomarkers selected from these kidney origin proteins for future validation should be linked with several different molecular functions and biological processes, thereby more comprehensively and accurately reflecting pathological conditions.

In the molecular function category, 933 proteins were linked to at least one annotation term. A total of 802 (86%) proteins were annotated as “binding function” and 549 (59%) proteins as “catalytic activity function”. The molecules bound by these proteins were very diverse, including proteins, metal ions, nucleotides, cofactors, peptides, amino acids, RNA, ubiquitin, and ribosomes. Enzyme activity–related GO terms were overrepresented, including “hydrolase activity”, “peptidase activity”, “peptidase regulator activity”, “GTPase activity”, “oxidoreductase activity”, and “ligase activity”. Enzyme inhibitors that can regulate these enzyme activities were also enriched. The proteins annotated in each molecular function category are summarized in Table S3.

In the biological process category, 948 proteins were linked to at least one annotation term. A total of 740 proteins were annotated as “metabolic process”. There were 711 overrepresented terms, which were mainly categorized into groups including “metabolic process”, “response to stimulus”, “transport”, “signaling and cell communication”, “gene expression”, and “protein modification process”. The proteins annotated in each biological process are summarized in Table S3.

## Discussion

This study aimed to identify human kidney origin proteins in urine. We present an approach to profile the isolated rat kidney perfusion-driven urine proteome, to match the identified rat proteins to human orthologs, and then we compare the human orthologs with human kidney expression data, the human urine proteome (urinary exosome proteome), and the plasma proteome. We also compared the perfusion-driven urine proteomes during perfusion with and without oxygen supplementation. Finally, we identified 990 human orthologs that were potential human kidney origin proteins in urine. We identified 428 high-quality kidney origin proteins that may become kidney disease biomarkers. These kidney origin proteins are either not present in plasma or normal urine or increased during perfusion. The kidney origin proteins identified in this study can be used to direct targeted proteomics studies in the discovery phase for kidney disease biomarkers. We recommend that the high-quality kidney origin proteins be screened first using targeted proteomics.

Isolated organ perfusates have advantages in the search for potential biomarkers, including accessibility, sensitivity and specificity. Many proteins that are differentially expressed in tissue are not detectable in bodily fluids. Perfusates are a reflection of the proteins that are accessible in bodily fluids. The concentration of the potential biomarkers is higher in perfusates than in bodily fluids. When compared with plasma or urine, perfusates reduce the proteome complexity to facilitate protein identification. Furthermore, perfusates exclude the influence of other organs. It should be noted that, a biomarker will only be fully relevant once it can be validated in the whole organism. The candidate biomarkers identified in organ perfusates should be validated in bodily fluids relevant to the disease condition of the specific organ.

To maintain physiological kidney function, an oncotic agent was included in the perfusates to create “physiological” colloid osmotic pressure. In previous studies, BSA was the most commonly used oncotic agent. However, it was not appropriate for this study because high amounts of BSA would be filtered into the urinary tract and interfere with the detection of kidney origin proteins. Dextran was used as an oncotic agent in this study. As a result, many protein preparation methods could not be used because dextran is readily precipitated by ethanol, acetonitrile, and acetone and would be retained by the reversed-phase chromatography column. However, dextran does not affect SDS-PAGE, which makes in-gel digestion of the proteins for LC-MS/MS analysis possible.

## Supporting Information

Table S1 The identified proteins in the isolated rat kidney perfusion-driven urine.
(XLS)

Table S2 The total human kidney origin proteins in urine.
(XLS)

Table S3 The human kidney origin proteins in urine were classified in terms of molecular function and biological process.
(XLS)
